# Photostimulation of lymphatic clearance of β-amyloid from mouse brain: a new strategy for the therapy of Alzheimer’s disease

**DOI:** 10.1007/s12200-023-00099-8

**Published:** 2023-12-14

**Authors:** Dongyu Li, Hao Lin, Silin Sun, Shaojun Liu, Zhang Liu, Yuening He, Jingtan Zhu, Jianyi Xu, Oxana Semyachkina-Glushkovskaya, Tingting Yu, Dan Zhu

**Affiliations:** 1 Britton Chance Center for Biomedical Photonics - MoE Key Laboratory for Biomedical Photonics, Wuhan National Laboratory for Optoelectronics - Advanced Biomedical Imaging Facility, Huazhong University of Science and Technology, Wuhan, 430074 China; 2 School of Optical Electronic Information, Huazhong University of Science and Technology, Wuhan, 430074 China; 3 Department of Biology, Saratov State University, Saratov, 410012 Russia

**Keywords:** Photostimulation, Meningeal lymphatic vessels, Amyloid-β clearance, Alzheimer’s disease

## Abstract

Alzheimer’s disease (AD) is an age-related neurodegenerative disorder that poses a significant burden on socio-economic and healthcare systems worldwide. However, the currently available therapy of AD is limited, and new strategies are needed to enhance the clearance of β-amyloid (Aβ) protein and improve cognitive function. Photobiomodulation (PBM) is a non-invasive and effective therapeutic method that has shown promise in treating various brain diseases. Here, we demonstrate that 1267-nm PBM significantly alleviates cognitive decline in the 5xFAD mouse model of AD and is safe as it does not induce a significant increase in cortical temperature. Moreover, with the combination of 3D tissue optical clearing imaging and automatic brain region segmentation, we show that PBM-mediated reductions of Aβ plaques in different subregions of prefrontal cortex and the hippocampus are different. The PBM-induced lymphatic clearance of Aβ from the brain is associated with improvement of memory and cognitive functions in 5xFAD mice. Our results suggest that the modulation of meningeal lymphatic vessels (MLVs) should play an important role in promoting Aβ clearance. Collectively, this pilot study demonstrates that PBM can safely accelerate lymphatic clearance of Aβ from the brain of 5xFAD mice, promoting improvement of neurocognitive status of AD animals suggesting that PBM can be an effective and bedside therapy for AD.

**Graphical Abstract:**

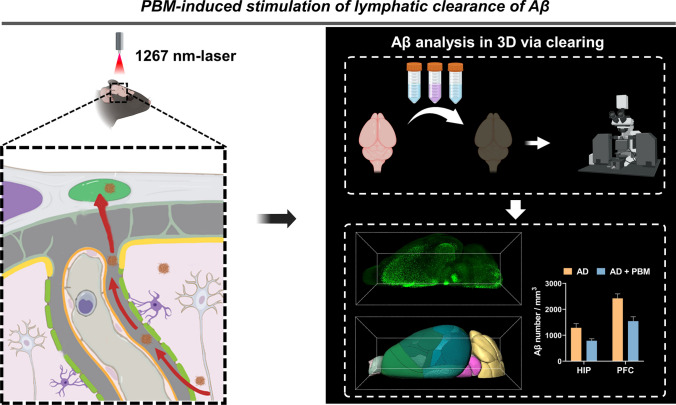

## Introduction

Alzheimer’s disease (AD) is an age-related neurodegenerative disorder. AD is typically characterized by obvious cognitive decline that can impair the ability to carry out daily activities, resulting in poor life quality and even death [1]. β-amyloid (Aβ) deposition in the brain is a crucial contributor to the pathogenesis of AD. The amyloid cascade hypothesis suggests that the deposition of Aβ peptide can lead to oxidative stress, neuroinflammation, neuronal loss, and cognitive deficits [2]. Therefore, mitigating excessive cerebral Aβ burden has been considered as a possible therapeutic strategy for AD [3].

The photobiomodultion (PBM), also known as low-level light therapy, refers to applying low irradiance (0.01–10 W/cm^2^) red to near-infrared (NIR) (600–1300 nm) light to cells and tissues, expecting to achieve neuroprotective, and behavioral improvement [4, 5]. There is compelling evidence suggesting that PBM can ameliorate Aβ burden in the brain of animal models of AD via various physiological processes and signals. Tao et al. demonstrated 2-month LED treatment at 1070 nm attenuated Aβ burden by modulating microglia phagocytosis capacity and promoting angiogenesis in AD mice [6]. Li et al. revealed that far infrared (3–25 μm) LED treatment lasting 1.5 months can enhance Aβ phagocytosis of microglia in AD mice via PI3K/mTOR pathways [7]. Yang et al. highlighted the importance of early prevention and interventions for AD treatment; they administered 808-nm laser to AD rats, and found PBM enhanced recruitment of microglia surrounding Aβ plaques by improving the expression of microglial IL-3Rα and astrocytic IL-3 in AD rats [8]. In addition, Xing’s lab proposed that 633-nm laser treatment for 1 month shifted the amyloid precursor protein processing toward the nonamyloidogenic pathway [9]. Tong et al. showed that the use of a 630-nm PBM treatment for 2 months could reverse interstitial fluid (ISF) flow obstruction and promote Aβ clearance in the brain of AD mice [10]. Meningeal lymphatic vessels (MLVs) are recently discovered structures responsible for exchanging soluble components between the cerebrospinal fluid (CSF) and ISF, and have been proved to be another pathway of Aβ drainage [11]. Recent works reported that PBM with 1267 nm laser could accelerate lymphatic clearance of exogenous Aβ from the brain into the deep cervical lymph nodes (dcLNs) [12, 13], indicating that the effects of PBM on AD may also be related to the stimulation of MLVs. However, the disease model with exogenous Aβ may not fully replicate the complexities of real pathology, hence the effect of PBM on the lymphatic clearance of endogenously produced Aβ remains uncertain. Moreover, it remains unclear whether PBM exhibits variations in enhancing Aβ efflux in different brain regions.

In this pilot study, we investigated the therapeutic effects of 1267-nm PBM in 5xFAD mice focusing on PBM-mediated stimulation of lymphatic clearance of Aβ from the different brain regions. First, we studied the safety effects of PBM by investigating the impact of different PBM doses on the temperature of the cortex surface, and analyzed the effectiveness of different PBM doses for improvement of cognitive functions. Then, we performed western blot analysis to measure the PBM-induced clearance of Aβ in the whole brain, combined with tissue optical clearing imaging, brain atlas registration and brain region segmentation technologies, we compared the effects of PBM on subregions of the prefrontal cortex and the hippocampus by quantitatively analyzing the reduction of Aβ density. Finally, we investigated the effects of PBM on AD brain lymphatic system by measuring the diameter of MLVs and evaluating their ability to drain tracers from the brain to dcLNs.

## Materials and methods

### Animals

**Fig. 1:**
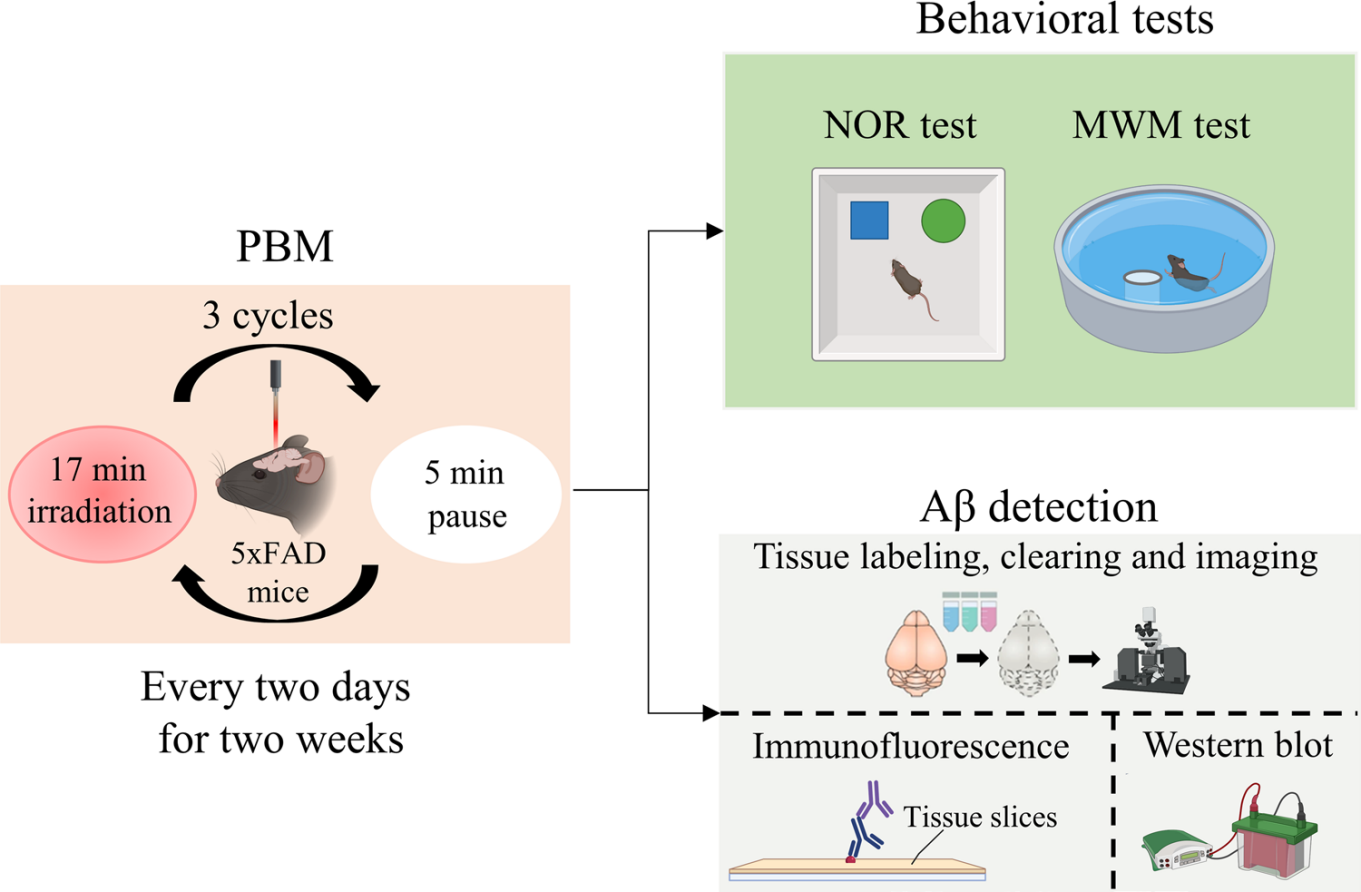
Experimental design. The male 5xFAD mice were subjected to PBM treatment, followed by behavioral tests or Aβ detection

Male heterozygous 5xFAD mice and C57BL/6 J wild-type (WT) mice (4–6 months old) weighting 26–28 g were used in our study. They were housed in an animal room under standard laboratory conditions, with access to water and food, ad libitum. Male homozygous 5xFAD mice [14] were initially purchased from the Jackson Laboratory in the United States and bred with 8-week-old female C57BL/6 J mice (purchased from the Experimental Animal Center of Wuhan University). The offspring were genetically identified and used for the experiments. All animal procedures were approved by the Experimental Animal Management Ordinance of Hubei Province, China, and carried out in accordance with the guidelines for humane care of animals. The specific experimental procedures are shown in Fig. 1.

### PBM treatment

A commercially available laser diode (LD-1267-FBG-350, Innolume, Dortmund, Germany) emitting at 1267 nm was used for irradiation sessions. The mice’s heads were shaved and the mice were fixed in a stereotaxic frame under inhalation anesthesia (1% isoflurane at 1 L⁄min N_2_/O_2_—70/30 ratio). Laser radiation was focused into a 0.2-cm^2^ round spot with a single mode distal fiber ended by the collimation optics. The light spot was delivered transcranially on the region of the sagittal sinus. The AD mice received PBM sessions once every two days for two weeks with the sequence of 17 min—irradiation, 5 min—pause, 17 min—irradiation, 5 min—pause, 17 min—irradiation, 61 min in total on each session [13, 15]. Three laser fluences on the sagittal sinus were used at this study—16, 32, and 64 J/cm^2^.

The heating of the brain surface caused by exposure to laser was monitored by using a thermocouple system (CAIPUSEN, YET-620L, China). Specifically, the medial part of the left temporal muscle was detached from the skull bone, a small burr hole was drilled into the temporal bone, and a flexible thermocouple probe was introduced to the brain surface. A thermocouple data logger was used to record the temperature during PBM treatment.

### Morris water maze (MWM) test

A MWM test was performed to evaluate the cognitive improvements [16, 17]. Briefly, the apparatus is made up of circular water tank with 120 cm diameter and 50 cm height. The tank filled with water (22 ± 1 °C) to a depth of 30 cm was made opaque by adding white food additives. A transparent survival platform (10 cm in diameter, circular) was placed hidden 1 cm below the water surface in one quadrant of the tank. During the training sessions, the mice received four training periods per day, in different quadrants, for 5 consecutive days. A probe trial was conducted 24 h after the training (day 6) with the hidden platform removed. Each trial lasted 1 min, the escape latency from the water and swim speed were calculated. All the data were recorded via a visual tracking system (Supermaze, China).

### Novel object recognition (NOR) test

The NOR test, a common method for examining cognition, particularly recognition memory [16], was also used to determine the optimal light fluence. During a habituation session, mice were placed into the empty open field and allowed to freely explore for 10 min. After a 24 h period, two identical non-toxic objects were placed in opposite and symmetrical corners of the open field; each mouse was once again released into the open field and allowed free exploration for a 5-min period. One of the previously explored objects was replaced 24 h later by a novel object. The mice were returned to explore the open field for an additional 5 min to test preference for the novel object, which we quantified by a recognition index,

$${\text{Recognition index }} = {T_{{\text{novel}}}}/\left( {{T_{{\text{novel}}}} + {T_{{\text{familiar}}}}} \right),$$

where* T*_novel_ and *T*_familiar_ indicate the exploration time during testing for the novel and familiar objects, respectively.

### Western blot analysis

A western blot analysis was performed following the protocol described in a previous study [15]. The total protein of each brain sample was measured by the correction in bicinchoninic acid protein assay kit (Thermo Scientific, Rockford, USA). The protein from each sample was separated by 4%–12% NuPAGE (180-8018H, Tanon). After transfer of proteins to polyvinylidene fluoride membrane (Millipore, Billerica, USA), the membrane was blocked with 5% skim milk for 1.5 h at room temperature and incubated for 2 h at room temperature with the primary antibodies: anti-Aβ (1:1000; D54D2, Cell Signaling Technology, USA). After that, the membrane was incubated with HRP-conjugated secondary antibody at room temperature for 1 h and treated with enhanced chemiluminescent reagent kit (Thermo Scientific, Rockford, USA). The bands were scanned and digitalized, the density of each band was quantified using FIJI software and normalized to the values of β-actin.

### Immunostaining and clearing

The mice were sacrificed by cervical dislocation to obtain the dcLNs, and cardiac perfusion was performed to obtain the meninges and brains. Subsequently, the obtained tissues were postfixed in 4% PFA solution at 4 °C overnight, followed by PBS rinse.

To visualize the lymphatic system in meninges, the whole-mount meninges were incubated in the blocking solution (a mixture of 0.2% Triton-X-100 and 10% normal goat serum in PBS) for 4 h, followed by incubation with Alexa Fluor 488-conjugated anti-Lyve-1 antibody (1:500; FAB2125G, R&D Systems, USA) and rabbit anti-Prox-1 antibody (1:500; ab101851, Abcam, United Kingdom) overnight at room temperature. After washing, the meninges were incubated with goat anti-rabbit IgG (H + L) Alexa Fluor 561 (Invitrogen, USA).

To visualize the Aβ in dcLNs, the dcLNs were fixed in 2% agarose, and sliced into 100 μm-thick sections using a vibratome (Leica VT1000, Germany). Then, the sections were incubated in the blocking solution (a mixture of 0.2% Triton-X-100 and 10% normal goat serum in PBS) for 1 h, followed by incubation with Alexa Fluor 488-conjugated anti-Lyve-1 antibody and rabbit anti-Aβ antibody (1:500; D54D2, Cell Signaling Technology, USA) overnight at room temperature. After washing, the sections were incubated with goat anti-rabbit IgG Alexa Fluor 555 secondary antibody (1:500; Invitrogen, USA).

For the brain, the iDISCO + method [18] was adopted for whole-mount immunostaining and tissue clearing. After pre-treatment with methanol, the brains were incubated in the blocking solution (a mixture of 0.2% Triton-X-100 and 10% normal goat serum in PBS) for 2 h, followed by incubation with anti-Aβ antibody (1:500; D54D2, Cell Signaling Technology, USA) for 7 days at 37 °C. After washing, the brains were incubated with goat anti-rabbit IgG Alexa Fluor 555 secondary antibody (1:500; Invitrogen, USA) for 7 days at 37 °C.

For tissue clearing, the samples were dehydrated with gradient methanol solutions (M116115, Aladdin, China) at concentrations of 20%, 40%, 60%, 80%, 100%, 100% (vol/vol) followed by incubation in the mixture of dichloromethane and methanol (2:1, vol:vol) overnight. After washing with 100% dichloromethane, the samples were transferred to dibenzyl ether (108014, Sigma-Aldrich, USA) until they became transparent. During clearing, the samples were kept in dark conditions.

### Intracerebral injection of Evans blue dye (EBD)

Anesthetized mice were fixed on a stereotactic apparatus, the skull was exposed, and a small burr hole was made over the right lateral (AP = − 0.5 mm, ML = − 1.06 mm). 3 µL of 0.2% EBD (Sigma-Aldrich) was injected (0.5 mL/min) into the right lateral ventricle to a depth of 2.5 mm. And 20 min later, the ventral skin of the neck was cut and the dcLNs were exposed.

### Imaging and image analysis

For imaging of immunostained meninges and dcLNs slices, the confocal laser scanning microscope (LSM710, Carl Zeiss, Germany) was adopted. For whole-brain imaging of cleared samples, a commercial light-sheet microscopy (LiToneXL, Light Innovation Technology, China) was used. To monitor brain lymphatic drainage in vivo, the stereo fluorescence microscope (Axio Zoom. V16, Zeiss, Jena, Germany) was used for imaging of dcLNs.

All fluorescent images were analyzed using FIJI software or Imaris v7.6 software (Bitplane AG, Switzerland).

For measurement of the meningeal lymphatic vessel diameter, a pipeline described in our previous study [15] was performed. Briefly, Otsu’s method was used to obtain the binary image. Then, morphological processes were performed and the profile curve was determined. After that, the diameter distribution of lymphatic vessels was calculated. By choosing two points on each side of a lymphatic vessel, a line between the two points to which tangents were perpendicular, could be taken as the diameter at this location. Following the above rules, the diameter of lymphatic vessels at each location could be obtained.

Quantification of Aβ in indicated brain regions was performed after brain tissue labeling, clearing (as described in Sect. 2.6) and imaging. ClearMap [19] was used for brain atlas registration first. In brief, the deformation field was obtained by aligning the 10 μm CCFv3 atlas to the acquired spontaneous fluorescence images. Then, the deformation field was applied to the atlas labels, resulting in the wrapped labels. The Aβ signal in specific brain regions was obtained via mapping the Aβ channel signal onto different brain regions under the indication of the wrapped labels. Then, the Aβ density in indicated regions was determined with Imaris.

### Statistical analysis

All data were presented as mean ± standard deviation. Statistical analysis of the data was conducted in GraphPad Prism 9. *p* values were calculated using one-way ANOVA or *t*-test. All *p* values < 0.05 were considered statistically significant.

## Results

### Effects of different doses of PBM on temperature of the cortex and cognitive functions in 5xFAD mice

**Fig. 2:**
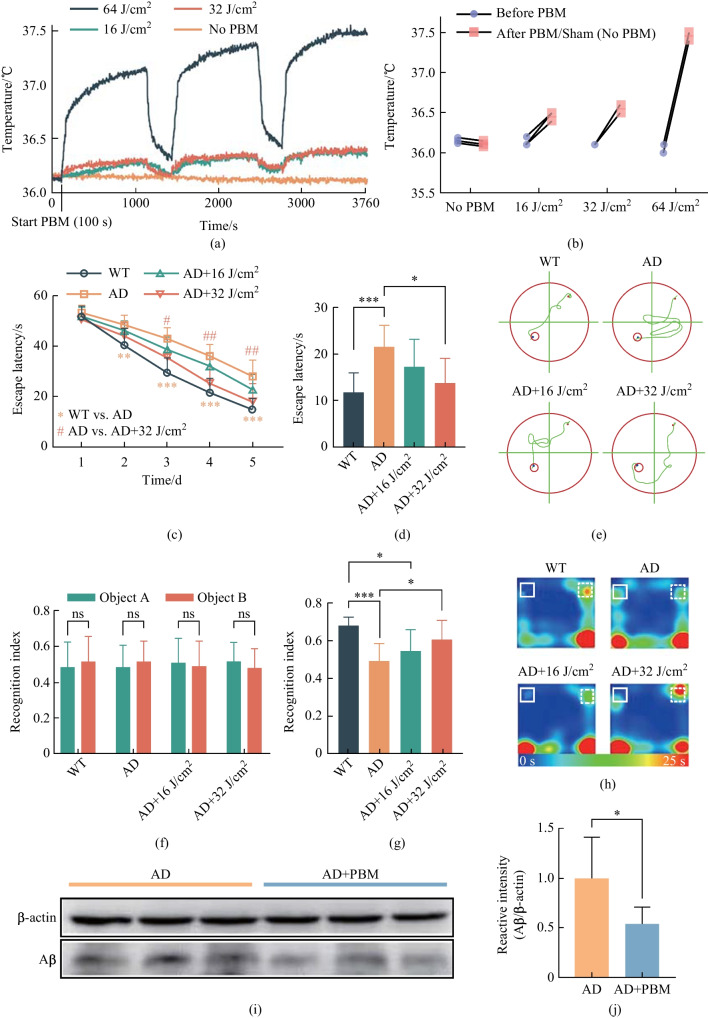
Effect of different doses of PBM on the changes of temperature on the cortex surface, cognitive functions and the Aβ level in mouse brain.** a** Average temperature change in the brain’s surface during PBM with different doses. **b** Maximum temperature difference of individual mouse during PBM treatment with different dose. **c** Escape latency of mice during MWM training session before and after different doses of PBM. **d** Escape latency and **e** typical swimming path in probe trial on day 6. The recognitive index in training **f** and testing **g** sessions in NOR test. **h** Typical movement paths on the testing session in NOR test. **i** Western blot representative image. **j** Quantitative analysis of Aβ in the brain. Notes: Data in **c**, **d**, **f**, **g** and **j** are presented as mean ± standard deviation,* n* = 3 for **a**,* n* = 8 for **c**, **d**, **f** and **g**, *n* = 10 for **j**, *p < 0.05, **p < 0.01, ***p < 0.001

In the first step, we studied the safety effects of different doses of PBM focusing on the analysis of the changes in temperature on the cortex surface using fluences of 16, 32 and 64 J/cm^2^. As shown in Fig. 2a and b, temperature increases on brain surface during the 16, 32 and 64 J/cm^2^ PBM treatments were 0.37 °C, 0.42 °C and 1.52 °C, respectively. Considering that 0.5 °C increase of cortical temperature could cause irreversible thermal damage such as meninges vasogenic edema and brain neuronal damage [13, 20], only lower PBM dosages of 16 and 32 J/cm^2^ were used in the following effectiveness tests.

In the second step, we investigated the PBM effects on the neurocognitive status of 5xFAD mice using the MWM and NOR tests. Figure 2c–e show that the 5xFAD mice displayed longer escape latencies at day 2, 3, 4 and 5 compared with the WT mice; PBM significantly improved escape latency and 32-J/cm^2^ PBM showed stronger therapeutic effect than 16-J/cm^2^ PBM (Fig. 2c). Moreover, on the day 6 probe trial, the hidden platform was removed, our results showed that the mice in 32-J/cm^2^ PBM group found the survival platform faster with a shorter swimming path than the case for AD and 16-J/cm^2^ PBM groups (Fig. 2d and e); there were no significant differences in average swimming speed among four groups, indicating the improvement of escape latency was not due to the differences in swimming speed. Because the dose of PBM 32 J/cm^2^ was more effective in performing the MWM test, the same dose was used to evaluate the NOR test for the study of the spatial learning and recognition memory (Fig. 2f–h). During the training session, there was no significant difference in recognition index among four groups (Fig. 2f). However, in the testing session, the recognition index significantly declined in the AD mice compared with the WT mice, and this effect was significantly improved by administration of PBM at 32 J/cm^2^ (Fig. 2g and h). In addition, the average velocity and total distance traveled did not differ significantly among four groups, indicating that the higher recognition index was not due to general differences in activities.

Thus, the results of these series of experiments clearly demonstrate that 1267-nm PBM at 32 J/cm^2^ has better effects on the neurocognitive status of 5xFAD mice than PBM at 16 J/cm^2^. Therefore, we used 32-J/cm^2^ PBM in the next steps of our study.

### PBM-mediated stimulation of clearance of Aβ from the brain of 5xFAD mice

**Fig. 3:**
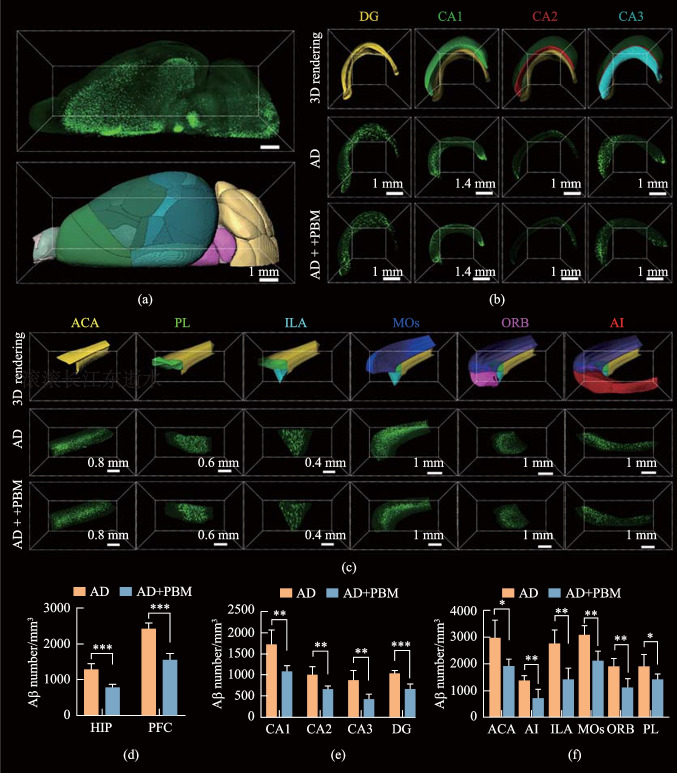
PBM-induced Aβ clearance from the prefrontal cortex and the hippocampus. **a** 3D reconstruction of Aβ in the brain hemisphere after tissue labeling, clearing and imaging, as well as its rendering image after registration and segmentation. **b** Representative 3D fluorescent images of Aβ in the hippocampus DG, CA1, CA2 and CA3. **c** Representative 3D fluorescent images of Aβ in the prefrontal cortex ACA, PL, ILA, MOs, ORB and AI. **d** Clearance of Aβ plaques in the prefrontal cortex and the hippocampus. **e** Clearance of Aβ plaques in DG, CA1, CA2 and CA3. **f** Clearance of Aβ plaques in ACA, PL, ILA, MOs, ORB and AI. Data are presented as mean ± standard deviation (*n* = 5), *p < 0.05, **p < 0.01, ***p < 0.001

The results of western blot analysis revealed that the overall Aβ levels in the whole brain of AD mice were mitigated after PBM treatment (Fig. 2i and j). Therefore, in the next step, we studied the PBM effects on the Aβ clearance from the different subregions of the prefrontal cortex and the hippocampus. By combining tissue optical clearing imaging (iDISCO +) with ClearMap brain atlas registration and brain region segmentation technologies, we investigated the Aβ clearance in specific subregions of the prefrontal cortex and hippocampus, which play important roles in attention, language, memory, etc. (Fig. 3). Our results showed that the density of Aβ plaques in the prefrontal cortex was higher than that in the hippocampus in 5xFAD mice. After PBM treatment, the density of Aβ plaque was significantly reduced (*p* < 0.001), and the reduction was statistically similar between the prefrontal cortex and the hippocampus (for the prefrontal cortex: reduction rates = 39%; for the hippocampus: reduction rates = 36%) (Fig. 3d). Further analysis showed that the density of Aβ plaque decreased in all subregions of the prefrontal cortex and the hippocampus with brain region specificity (Fig. 3b, c, e and f). Specifically, PBM reduced by 37%, 34%, 51%, and 36% Aβ plaques in hippocampus CA1, CA2, CA3 and dentate gyrus (DG), respectively. These brain regions play important roles in long-term memory, social memory, spatial memory, memory retention and similar memories discrimination [21–23].

The medial prefrontal cortex, including the anterior cingulate area (ACA), prelimbic area (PL), infralimbic area (ILA), and secondary motor area (MOs), play crucial roles in cognitive process and emotion regulation [24]. The density of Aβ plaques in these four brain regions was decreased by 36%, 26%, 48% and 31% after PBM, respectively. The orbital area (ORB) and agranular insular area (AI) belong to lateral prefrontal cortex, which is involved in feeling, cognitive function, social behavior and decision making [25, 26]. The PBM decreased by 42% and 49% Aβ plaques in above two brain regions, respectively.

Overall, after PBM, the overall Aβ deposition in the brain was significantly reduced. 3D data indicated that the densities of Aβ plaques in the prefrontal cortex and in the hippocampus subregions associated with memory and cognitive function were all significantly reduced, to varying degrees, corresponding with the results of the MWM and NOR tests.

### PBM-induced stimulation of lymphatic clearance of Aβ

**Fig. 4:**
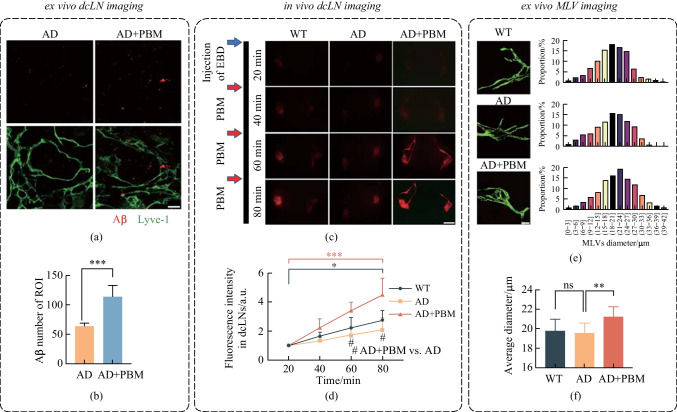
PBM effects on lymphatic clearance of Aβ from the brain of 5xFAD mice. **a** Representative fluorescent images and **b** statistical image of Aβ plaques in dcLNs. Scale bar = 20 μm. **c** Representative fluorescent images of EBD clearance from the right lateral ventricle into the dcLNs in WT, AD and AD + PBM groups. Scale bar = 2 mm. **d** Quantitative analysis of fluorescence intensity of EBD in dcLNs in WT, AD and AD + PBM groups. **e** Representative fluorescent images and diameter distribution of basal MLVs (labelled with anti-LYVE-1 and anti-PROX-1) in WT, AD and AD + PBM groups. Scale bar = 50 μm. **f** Quantitative analysis of average diameter of the basal MLVs in WT, AD and AD + PBM groups. Notes: Data in **b, d** and **e** are presented as mean ± standard deviation, *n* = 10 for **b**, **e** and **f**, *n* = 3 for **d**, ns: not significant, *p < 0.05, **p < 0.01, ***p < 0.001

The above results show that PBM mitigated Aβ burden in the brain of 5xFAD mice. Previous study reported that the MLVs-dcLNs pathway is an important route for elimination of the metabolic wastes and toxins in the brain [11]. With the hypothesis that PBM could promote Aβ elimination in this way in 5xFAD mice, we first examined the Aβ levels in dcLNs by immunofluorescence staining. As shown in Fig. 4a and b, the quantity of Aβ was significantly increased in dcLNs after 14-day PBM (AD vs AD + PBM,* p* < 0.001). Then, we investigated the effects of PBM on stimulating the lymphatic clearance of EBD from the brain, with the aim of mimicking the process of Aβ elimination from the brain. Figure 4c and d show that AD was accompanied with a reduction of lymphatic removal of EBD from the brain and accumulation of dye in the dcLNs. These findings indicate deteriorated lymphatic clearance function in AD mice, which is consistent with the results of other studies [27]. However, after PBM, lymphatic clearance of EBD was significantly improved (AD vs AD + PBM, 60 min: *p* = 0.029; 80 min: *p* = 0.036). Moreover, we found PBM could increase the diameter of the basal MLVs (AD vs AD + PBM, *p* = 0.007), as shown in Fig. 4e and f. Above results suggested that PBM could improve the lymphatic drainage via modulation of tone of MLVs, promoting the Aβ clearance from the brain into dcLNs in AD mice.

## Conclusion and discussion

In the present study, we demonstrate that 1267-nm PBM at 32 J/cm^2^ can significantly alleviate cognitive decline in 5xFAD mice without an obvious temperature increase in the brain’s surface. In addition, we propose that the 1267-nm PBM can improve the lymphatic drainage via relaxation of MLVs, thus promoting Aβ clearance from the brain into dcLNs. Moreover, 3D results show that the density of Aβ plaques in all subregions of the prefrontal cortex and the hippocampus is decreased. Thus, our data corroborate that 1267-nm PBM may be a promising therapeutic strategy for AD treatment.

The amyloidosis is one of the reasons for AD-associated neurodegeneration and cognitive impairment [28, 29]. Therefore, determining the Aβ spatial distribution should be critical for revealing the pathogenic mechanism and for illustrating pathological manifestations of AD, as well as being convenient for screening and examining the effectiveness of therapeutic methods. A combination of transgenic AD mice, tissue clearing and labeling method, as well as brain atlas registration and segmentation method, together provides a potential strategy to illuminate the Aβ plaques in the whole-mount brain. The hippocampus regions CA1 and CA3 are known to play crucial roles in spatial memory and consolidation of long-term memory, both of which are adversely affected in the early stages of AD. Our findings indicate that 1267-nm PBM leads to significant reduction of Aβ plaques in CA1 and CA3 by 37% and 51%, respectively. These results are consistent with the outcomes of the MWM test. Moreover, for the hippocampus region DG responsible for the encoding, retrieval and discrimination of similar memories, we demonstrate a notable 36% decrease in the density of Aβ plaques in this brain region, consistent with the results of the NOR test. In addition, our data suggest that after PBM treatment, there was a significant reduction in the density of Aβ plaques across the six subregions of prefrontal cortex, albeit to varying degrees. Such results indicate that PBM may have the potential to enhance the functioning of these cognitive processes, which could be further investigated in the future studies.

The MLVs system provides an efficient pathway for Aβ efflux from the brain, and has been proved as a potential target for AD treatment. Previous studies have presented compelling evidence for the effectiveness of PBM in AD treatment [6, 10]. However, the study of PBM effects on MLVs drainage and lymphatic clearance of Aβ from the brain as a promising method for the AD therapy is in its infancy [13]. Our findings in the change of the basal MLVs diameter demonstrate PBM-modulation of the MLVs functions can play an important role in regulating the clearance of Aβ. This is consistent with our previous data suggesting that PBM can increase the diameter of MLVs contributing to lymphatic clearance of blood from the brain of mice with intraventricular hemorrhages [30]. Additionally, in our another work [15], we have observed that this PBM treatment can improve the morphology and reactivity of cortical microglia in diabetic mice, and the activation of the brain lymphatic system contributing to the removal of inflammatory factors, like interferon gamma from the brain parenchyma, appears to be a significant factor in the observed behavioral improvement [15]. Indeed, PBM-mediated biological effects have recently been identified to involve multiple physiological processes such as preservation of mitochondrial dynamics and regulation of redox metabolism [8, 31]. Whether our PBM method has such biological effects merits further in-depth investigation in future studies.

In summary, our results demonstrate that 1267-nm PBM is a non-invasive and effective therapeutic approach in mitigating cerebral Aβ burden and alleviating cognitive decline of 5xFAD mice. PBM-mediated stimulation of Aβ elimination from the brain via the lymphatic pathway may be a key mechanism in the therapeutic effects of PBM for AD in mice. In addition, the research method we propose here could be a powerful tool in the study of AD.

## Data Availability

The data that support the findings of this study are available from the corresponding author, upon reasonable request.

## References

[1] Scheltens P, De Strooper B, Kivipelto M, Holstege H, Chételat G, Teunissen CE, Cummings J, van der Flier WM (2021). Alzheimer’s disease. Lancet.

[2] Hardy JA, Higgins GA (1992). Alzheimer’s disease: the amyloid cascade hypothesis. Science.

[3] Cheng Y, Tian D, Wang Y (2020). Peripheral clearance of brain-derived Aβ in Alzheimer’s disease: pathophysiology and therapeutic perspectives. Transl. Neurodegener..

[4] Salehpour F, Khademi M, Bragin DE, DiDuro JO (2022). Photobiomodulation therapy and the glymphatic system: promising applications for augmenting the brain lymphatic drainage system. Int. J. Mol. Sci..

[5] Farivar S, Malekshahabi T, Shiari R (2014). Biological effects of low-level laser therapy. J. Lasers. Med. Sci..

[6] Tao L, Liu Q, Zhang F, Fu Y, Zhu X, Weng X, Han H, Huang Y, Suo Y, Chen L, Gao X, Wei X (2021). Microglia modulation with 1070-nm light attenuates Aβ burden and cognitive impairment in Alzheimer’s disease mouse model. Light. Sci. Appl..

[7] Li Q, Peng J, Luo Y, Zhou J, Li T, Cao L, Peng S, Zuo Z, Wang Z (2022). Far infrared light irradiation enhances Aβ clearance via increased exocytotic microglial ATP and ameliorates cognitive deficit in Alzheimer’s disease-like mice. J. Neuroinflammation.

[8] Yang L, Wu C, Parker E, Li Y, Dong Y, Tucker L, Brann DW, Lin HW, Zhang Q (2022). Non-invasive photobiomodulation treatment in an Alzheimer Disease-like transgenic rat model. Theranostics.

[9] Zhang Z, Shen Q, Wu X, Zhang D, Xing D (2020). Activation of PKA/SIRT1 signaling pathway by photobiomodulation therapy reduces Aβ levels in Alzheimer’s disease models. Aging Cell.

[10] Yue X, Mei Y, Zhang Y, Tong Z, Cui D, Yang J, Wang A, Wang R, Fei X, Ai L, Di Y, Luo H, Li H, Luo W, Lu Y, Li R, Duan C, Gao G, Yang H, Sun B, He R, Song W, Han H, Tong Z (2019). New insight into Alzheimer’s disease: light reverses Aβ-obstructed interstitial fluid flow and ameliorates memory decline in APP/PS1 mice. Alzheimers Dement..

[11] Da Mesquita S, Papadopoulos Z, Dykstra T, Brase L, Farias FG, Wall M, Jiang H, Kodira CD, de Lima KA, Herz J, Louveau A, Goldman DH, Salvador AF, Onengut-Gumuscu S, Farber E, Dabhi N, Kennedy T, Milam MG, Baker W, Smirnov I, Rich SS, Benitez BA, Karch CM, Perrin RJ, Farlow M, Chhatwal JP, Holtzman DM, Cruchaga C, Harari O, Kipnis J, Dominantly Inherited Alzheimer N (2021). Meningeal lymphatics affect microglia responses and anti-Aβ immunotherapy. Nature.

[12] Semyachkina-Glushkovskaya O, Penzel T, Blokhina I, Khorovodov A, Fedosov I, Yu T, Karandin G, Evsukova A, Elovenko D, Adushkina V, Shirokov A, Dubrovskii A, Terskov A, Navolokin N, Tzoy M, Ageev V, Agranovich I, Telnova V, Tsven A, Kurths J (2021). Night photostimulation of clearance of beta-amyloid from mouse brain: new strategies in preventing Alzheimer’s disease. Cells.

[13] Zinchenko E, Navolokin N, Shirokov A, Khlebtsov B, Dubrovsky A, Saranceva E, Abdurashitov A, Khorovodov A, Terskov A, Mamedova A, Klimova M, Agranovich I, Martinov D, Tuchin V, Semyachkina-Glushkovskaya O, Kurts J (2019). Pilot study of transcranial photobiomodulation of lymphatic clearance of beta-amyloid from the mouse brain: breakthrough strategies for non-pharmacologic therapy of Alzheimer’s disease. Biomed. Opt. Express.

[14] Oakley H, Cole SL, Logan S, Maus E, Shao P, Craft J, Guillozet-Bongaarts A, Ohno M, Disterhoft J, Eldik LV, Berry R, Vassar R (2006). Intraneuronal β-amyloid aggregates, neurodegeneration, and neuron loss in transgenic mice with five familial Alzheimer’s disease mutations: potential factors in amyloid plaque formation. J. Neurosci..

[15] Liu S., Li D., Yu T., Zhu J., Semyachkina-Glushkovskaya O., Zhu D.: Transcranial photobiomodulation improves insulin therapy in diabetic mice: modulation of microglia and the brain drainage system. 10.21203/rs.3.rs-2607673/v1 (2023) 10.1038/s42003-023-05630-3 PMC10709608 38066234

[16] Martorell AJ, Paulson AL, Suk HJ, Abdurrob F, Drummond GT, Guan W, Young JZ, Kim DNW, Kritskiy O, Barker SJ, Mangena V, Prince SM, Brown EN, Chung K, Boyden ES, Singer AC, Tsai LH (2019). Multi-sensory gamma stimulation ameliorates Alzheimer’s-associated pathology and improves cognition. Cell.

[17] Shen Q, Liu L, Gu X, Xing D (2020). Photobiomodulation suppresses JNK3 by activation of ERK/MKP7 to attenuate AMPA receptor endocytosis in Alzheimer’s disease. Aging Cell.

[18] Renier N, Adams EL, Kirst C, Wu Z, Azevedo R, Kohl J, Autry AE, Kadiri L, Venkataraju KU, Zhou Y (2016). Mapping of brain activity by automated volume analysis of immediate early genes. Cell.

[19] Renier N, Adams EL, Kirst C, Wu Z, Azevedo R, Kohl J, Autry AE, Kadiri L, Umadevi VK, Zhou Y, Wang VX, Tang CY, Olsen O, Dulac C, Osten P, Tessier-Lavigne M (2016). Mapping of brain activity by automated volume analysis of immediate early genes. Cell.

[20] Bhattacharya M, Dutta A (2019). Computational modeling of the photon transport, tissue heating, and cytochrome C oxidase absorption during transcranial near-infrared stimulation. Brain Sci..

[21] Lana D, Ugolini F, Giovannini MG (2020). Space-dependent glia-neuron interplay in the hippocampus of transgenic models of β-amyloid deposition. Int. J. Mol. Sci..

[22] Mankin EA, Diehl GW, Sparks FT, Leutgeb S, Leutgeb JK (2015). Hippocampal CA2 activity patterns change over time to a larger extent than between spatial contexts. Neuron.

[23] Hainmueller T, Bartos M (2020). Dentate gyrus circuits for encoding, retrieval and discrimination of episodic memories. Nat. Rev. Neurosci..

[24] Le Merre P, Ährlund-Richter S, Carlén M (2021). The mouse prefrontal cortex: Unity in diversity. Neuron.

[25] Van Hoesen GW, Parvizi J, Chu C (2000). Orbitofrontal cortex pathology in Alzheimer’s disease. Cereb. Cortex.

[26] Shi T, Feng S, Wei M, Zhou W (2020). Role of the anterior agranular insular cortex in the modulation of fear and anxiety. Brain Res. Bull..

[27] Da Mesquita S, Louveau A, Vaccari A, Smirnov I, Cornelison RC, Kingsmore KM, Contarino C, Onengut-Gumuscu S, Farber E, Raper D, Viar KE, Powell RD, Baker W, Dabhi N, Bai R, Cao R, Hu S, Rich SS, Munson JM, Lopes MB, Overall CC, Acton ST, Kipnis J (2018). Functional aspects of meningeal lymphatics in ageing and Alzheimer’s disease. Nature.

[28] Liang X, Luo H (2021). Optical tissue clearing: illuminating brain function and dysfunction. Theranostics.

[29] Ossenkoppele R, Smith R, Ohlsson T, Strandberg O, Mattsson N, Insel PS, Palmqvist S, Hansson O (2019). Associations between tau, Aβ, and cortical thickness with cognition in Alzheimer disease. Neurology.

[30] Li D, Liu S, Yu T, Liu Z, Sun S, Bragin D, Shirokov A, Navolokin N, Bragina O, Hu Z, Kurths J, Fedosov I, Blokhina O, Dubrovski A, Khorovodov A, Terskov A, Tzoy M, Semyachkina-Glushkovskaya O, Zhu D (2023). Photostimulation of brain lymphatics in male newborn and adult rodents for therapy of intraventricular hemorrhage. Nat. Commun..

[31] Huang Z, Zhang Y, Ma X, Feng Y, Zong X, Jordan JD, Zhang Q (2023). Photobiomodulation attenuates oligodendrocyte dysfunction and prevents adverse neurological consequences in a rat model of early life adversity. Theranostics.

